# Engaging the Private Health Service Delivery Sector for TB Care in India—Miles to Go!

**DOI:** 10.3390/tropicalmed8050265

**Published:** 2023-05-04

**Authors:** Rakesh P. Suseela, Mohd Shannawaz

**Affiliations:** 1 Amity Institute of Public Health, Amity University, Noida 201303, India; 2 The Union South East Asia Office, New Delhi 110016, India

**Keywords:** private sector engagement, public–private partnership, public–private mix, standards of TB care

## Abstract

More than half of the people with TB in India seek care from the private sector, where suboptimal quality of care is a concern. Significant progress has been made over the last five years to expand the coverage and to involve more private sector providers in TB care under the National TB Elimination Program (NTEP) in India. The objective of this review is to describe the major efforts and the progress made with regard to the engagement of the ‘for-profit’ private health service delivery sector for TB care in India, to critically discuss this, and to suggest the way forward. We described the recent efforts by the NTEP for private sector engagement based on the literature, including strategy documents, guidelines, annual reports, evaluation studies, and critically looked at the strategies against the vision of partnership. The NTEP has taken a variety of approaches, including education, regulation, provision of cost-free TB services, incentives, and partnership schemes to engage the private sector. As a result of all these interventions, private sector contribution has increased substantially, including TB notification, follow-up, and treatment success. However, these still fall short of achieving the set targets. Strategies were focused more towards the purchase of services rather than creating sustainable partnerships. There are no major strategies to engage the diverse set of providers, including informal health care providers and chemists, who are the first point of contact for a significant number of people with TB. India needs an integrated private sector engagement policy focusing on ensuring standards of TB care for every citizen. The NTEP should adopt an approach specifically tailored to the various categories of providers. For meaningful inclusion of the private sector, it is also essential to build understanding and generate data intelligence for better decision making, strengthen the platforms for engagement, and expand the social insurance coverage.

## 1. Introduction

India accounted for 28% of incident TB cases and 32% of estimated TB deaths globally in 2021 [1]. India is committed to achieve the Sustainable Development Goals related to ending TB by 2025, five years ahead of the global target [2]. Even though free diagnostic and treatment care are provided under the National TB Elimination Program (NTEP), the National TB Prevalence Survey (NTPS) has revealed that half of the people with TB symptoms sought care from the private sector [3]. Analysis of the data regarding the sales of anti-TB drugs in India revealed that the treatment of TB is dominated by the private healthcare sector, with nearly 2/3rd of the market share [4,5]. With a large number of people with TB being managed by the private sector, there are concerns about the suboptimal quality of care; lack of systems for ensuring treatment adherence, patient support, and contact investigations; and high lost to follow-up rate, eventually raising the risk of drug resistance to TB [6,7,8,9].

The National Strategic Plan for TB Elimination in India (2017–2025) deemed it crucial to ensure that patients reaching the private sector receive timely and quality-assured diagnosis and treatment, protection from high out-of-pocket expenditure, other public health services such as management of co-morbidities and adverse drug reactions, contact investigation and disease prevention, counselling, adherence support and monitoring, nutritional support, and outcome reporting [2]. Over the last five years, significant progress has been made under the NTEP to expand the coverage and to involve more private sector providers in TB care. The current review attempts to narrate the major efforts and the progress made so far with regard to private health service delivery sector engagement for TB care in India, to critically discuss this, and suggest the way forward.

## 2. Methods

**Scope and definitions:** We used the operational definition provided by World Health Organization, which defined the private health sector as ‘the individuals and organizations that are neither owned nor directly controlled by governments and are involved in provision of health services’ [10]. We focused on ‘for-profit’ healthcare providers, including both formal and informal providers, because they are more numerous and difficult to engage. We included only health service providers rather than manufacturers or distributors of medical equipment, technologies, consumables, or drugs. We used the definition of private sector engagement as “the meaningful inclusion of private providers for service delivery in mixed health systems”, as defined by the WHO Advisory Group on the Governance of the Private Sector for Universal Health Coverage [11]. The definition is broad in order to capture all modalities for engaging the private sector, from informal collaborations to more formalized partnerships. In the context of health service delivery, there is also a growing consensus that “it is essential to re-frame private sector engagement as a partnership in health for shared health outcomes” [10]. The WHO describes partnership as a means to “bring together a set of actors for the common goal of improving the health of populations based on mutually agreed roles and principles based on the principles of relative equality between the partners, mutual benefits to the stakeholders, autonomy, accountability and mutual commitment to agreed objectives” [10]. We have used this as the vision of partnership against which we compared various initiatives.

**Review:** The sources used for this review included searches of central government websites (Ministry of Health, NTEP, National Health Mission) and included policy documents, strategy documents, operational plans, guidelines, annual reports, and evaluation studies. We also looked at PubMed and Google Scholar with the search string “Tuberculosis” AND “private” AND “India” for the period 2000 to 2022. We included only studies referring to for-profit providers. We described the recent efforts by the NTEP for private sector engagement by various domains of engagement. Various evolving models of private sector engagement within the country were also described. We attempted to look at the outcomes of various initiatives based on the available literature and compare the outcomes against the plan. We also attempted to critically look at the strategies and initiatives against the vision of partnership described above.

## 3. Results

### 3.1. Efforts by NTEP for Private Sector Engagement

India’s TB program had realized the need to engage the private sector nearly two decades ago and had initiated a number of initiatives for Public–Private Mix (PPM). The NTEP has taken a variety of approaches, including education, regulation, provisions of cost-free services, incentives, and partnership schemes, to engage with private sector. Major initiatives recently undertaken by the Government of India for the engagement of private health service providers are shown in Table 1. The domains of engagement were adapted from the strategy report of the WHO Advisory Group on the Governance of the Private Sector for Universal Health Coverage [11]. The scale of implementation of these initiatives is highly uneven within the country.

### 3.2. Mode of Providing Public Provision of Services to Private Sector

**Patient Provider Support Agencies (PPSA)**: In 2014, the TB program initiated a pilot project on Universal Access to TB Care (UATBC). In Mumbai (Maharashtra) and Patna (Bihar), the program utilized the services of a Private Provider Support Agency (PPSA), a third-party agency, to engage private-sector doctors who treat patients with TB and provide end-to-end services, including diagnosis, notification, patient adherence and support, and treatment linkages. A three to five times increase in TB case notifications was documented at these pilot sites [23]. In Mumbai, the total notification increased from 272/lakh in 2013 to 351/lakh in 2015, and in Patna, it increased from 80/lakh to 355/lakh. At 100% coverage, the commodity cost per case was estimated to be USD 58 in Patna and USD 67 in Mumbai, whereas the programmatic cost per case was estimated to be USD 33 and USD 34 for the Patna and Mumbai sites, respectively [24]. The learnings from these pilots were scaled up across the country as the Patient Provider Support Agency (PPSA), and implemented as part of Project JEET (Joint Effort for the Elimination of Tuberculosis) [25]. PPSAs, predominantly NGOs, take on the tasks of engaging with individual providers, notifying cases to the NTEP, and coordinating with the NTEP for free drugs and follow-up. PPSA activities are approved in the budget for 385 districts and are currently functional in 188 districts.

**Direct NTEP engagement approach**: This approach primarily uses the existing NTEP staff, such as district PPM coordinators, TB health visitors in urban areas, and senior treatment supervisors, to engage with the private sector and provide support to the patients reaching the private sector.

### 3.3. Other Models of Private Sector Engagement for TB Care Evolved in India

**STEPS (System for TB Elimination in Private Sector)**: STEPS evolved as a local solution to improve quality of care where the efforts to ensure STCI for patients reaching the private sector is led by the private hospital management [26]. STEPS has three components: (i) a private hospitals TB consortium, (ii) a coalition of medical professional associations at state and district levels, and (iii) a STEPS center in each private hospital. Establishing STEPS centers at all private hospitals is at the heart of STEPS. These centers act as a “single window” in private facilities to ensure that all people with presumptive or confirmed TB receive care as per the STCI. They also provide TB notification, linkage for public health actions including contact investigations, chemoprophylaxis to eligible households, HIV testing, domestic airborne infection control kits (washable reusable masks, disinfectant solution, spittoon, information leaflet), linkages to monthly direct benefit transfers of INR 500 (USD 7) and nutritional support, and treatment adherence support. STEPS centers are visualized as an “after-sales service model” based on self-initiated business promotion and customer loyalty blended with the social responsibility of the private sector. In business, after-sales service is any support provided to a customer after the product or service has already been purchased. Companies use after-sales support as a business strategy because it typically leads to higher customer satisfaction, brand loyalty, and even word-of-mouth marketing. The STEPS model is in place in the major hospitals of the southern state of Kerala and a few cities in Karnataka and Tamilnadu. An evaluation revealed that STEPS is a low-cost and patient-centric strategy which successfully addressed the gaps in the quality of care for patients seeking care in the private sector hospitals [27].

**Private Provider Incentive Scheme (PPIS):** In the state of Rajasthan, in addition to the incentives for TB notification and reporting outcomes, other incentives are being provided to private practitioners for offering services to patients as per the STCI, such as drug susceptibility testing, HIV testing, co-morbidity screening, contact investigations, and TB preventive therapy. In total, 6764 private providers were engaged over the last two years who contributed to 25% of the total TB notification in the state. However, no formal evaluation of this model is available [28].

### 3.4. Outcomes of Private Sector Engagement

As a result of all these interventions, private sector notifications have increased substantially, and the quality of their TB services, including treatment success rates, has improved over the past five years. The trends in TB notification and related indicators for the patients notified from the private health care sector in India are shown in Table 2.

It was difficult to attribute how much each individual component has contributed to improvements in the indicators for private sector engagement.

There is no information available to compare the trend in out-of-pocket expenditure for TB diagnosis and treatment. The National TB Prevalence Survey has reported the total median (range) out-of-pocket cost for patients for the diagnosis and treatment of TB in the private sector as INR 16000 (2000–33,000) in comparison to INR 9000 (2000–33,000) in the public sector (USD 1 = INR 85) [3]. A review of studies conducted in India showed that a catastrophic cost (total cost ≥ 20% of the total annual household income) was experienced by 7% to 32.4% of drug-sensitive TB patients and by 68% of drug-resistant TB patients [30].

Global Burden of Diseases collaborators attempted to measure the universal health coverage based on an index of effective coverage of TB services; however, there was no data available to compare the trend [31].

### 3.5. Achievements in Comparison to the NSP Targets

**Figure 1:**
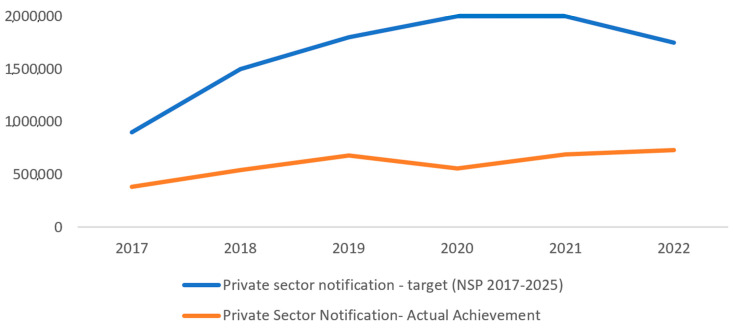
TB notification from the private sector in India-target vs. achievement (2017–2022) [2,29].

The NSP intended to achieve a target of 2 million TB notifications from the private sector in 2021; there is a gap of 1.3 million TB cases that are ‘missing’ in the surveillance system. The NSP envisioned to obtain 56% of the total notifications from the private sector by 2021; the country has reached only 32% [2,29]. In light of the very ambitious 2025 goals, the treatment success rate of people with TB under private care has improved over the past few years; however, it is still 8% below the target of 90%. The comparison of target vs. achievement related to TB notification from the private sector is shown in Figure 1.

### 3.6. Critical Observations

In spite of the progress in private sector engagement, major challenges continue to impede the pace of reaching out to all TB patients in the private sector. Major observations regarding the strategies and initiatives compared against the ‘vision of partnership’ are as follows:

- The NTEP guidance document on partnership talks in detail about the purchase of services but is silent about partnerships [20]. Most of the purchases of services by the NTEP were from the ‘not-for-profit’ health sector than from the ‘for-profit’ health care service delivery sector [15].
- Notifications and ensuring public health actions for people with TB have not been converted into routine practice in the private sector. Most of the partnership models in the country were incentive-based or service-purchase models that were similar to a client–vendor relationship. Several models that have successfully increased private case notifications were difficult to sustain and expand due to a lesser emphasis on creating sustainable partnerships.
- The number and characteristics of private providers is not clearly understood by the NTEP. The private sector is highly fragmented, ranging from informal health care providers to corporate hospitals. It also consists of providers of variable quality. A uniform strategy for engagement might not be relevant to all parties. In many states of India, informal health care providers outnumber formal health care providers [32,33]. They include village practitioners, drug sellers, untrained allopathic providers, traditional healers, and faith healers. They are the most frequent first port of call for rural residents seeking health care [34,35,36]. Patients approaching an informal health care provider currently experience delays in the diagnosis of TB [37,38,39]. To address the issue of delays in the diagnosis and to find out all the ‘missing cases’, it is essential to engage these informal providers. Similarly, the Ayurveda, Unani, Siddha, Homeopathy (AYUSH) providers and chemists in India are among the first point of care in several part of the country. There are no major strategies by the NTEP to engage with such a diverse set of providers.
- Public sector staff have limited capacity and skills to engage with the private sector [40]. For successful engagement of the private sector, public sector staff require a thorough understanding of the private sector system and require skills for advocacy, marketing, and communication. There is no mechanism to systematically impart such knowledge and skills to NTEP staff.
- There are no formal stakeholder platforms where the ‘for-profit health sector’ is present, limiting the level of dialogue between the NTEP and the private sector for any policy discussions.
- Though there is regulation, its poor implementation is a major challenge. Effective enforcement of this regulation is highly uneven, limited to a few locations. The drug control department finds it difficult to implement the Schedule H1 regulation due to inadequate human resources, and chemists find it difficult to document in their busy schedules [2,41].
- There are around 2000 private medical laboratories accredited by the National Accreditation Board for Testing and Calibration Laboratories (NABL) [42]. However, the efforts to extend TB laboratory services in the private sector are limited. There are 75 NTEP-certified culture and DST labs in the public sector, whereas there are only 17 in the private sector [15].
- The coverage in PM-JAY is currently limited to the indoor management of patients with pleural, pericardial, and neuro-tuberculosis [21].
- There are only a few studies with any in-depth follow-up on the process of the partnership or any longitudinal study of a partnership.

## 4. Discussion

The current review is an attempt to compile and describe the recent efforts by the NTEP for private sector engagement. Various evolving models of private sector engagement within the country were described, and outcomes of various initiatives based on the available literature and a comparison of the outcomes against the plan were described. Such a compilation will provide a comprehensive picture regarding the engagement of the private sector in TB care in India.

However, due to the non-availability of systematic studies, it was difficult to estimate which strategy contributed to what percentage of mobilization so far of the private sector for TB care. Additionally, we could not ascertain the level of implementation of each strategy. We also could not perform a detailed cost analysis of how much the country has spent on private sector engagement, as the information is not available in the public domain.

Based on the analysis, we suggest the following as the way forward.

### Suggestions for the Way Forward

**Developing strategic policy direction**: Based on the learnings and developments over the last few years, there is a need to redefine the strategic policy direction for private sector engagement. In a country such as India, where the resources are limited and the private sector is huge, it would be ideal to reframe ‘private sector engagement for TB care’ as ‘partnership for TB elimination’, where both the public and private sector parties work together towards the betterment of society with a common goal of TB elimination. Such a partnership could be viewed as a relationship and not as a mere contract. More options for creating lasting partnerships with the private health sector and a mechanism to ensure the accountability of both sectors need to be brought in. Having a clear policy and strategy will help to avoid confusion among the stakeholders and will enable them to perform better. It should clearly define the goals and objectives of private sector engagement, clarify roles of all stakeholders, describe the institutional arrangements for engagement, and outline the feasible strategies and arrangements to monitor performance.

**Build understanding and generate data intelligence:** Knowing the denominator is really important for planning the efforts required and monitoring the progress with respect to the engagement of the private sector. The routine monitoring of sales of anti-TB drugs at the district level could help in estimating the burden of TB patients treated with anti-TB drugs outside the NTEP and understanding its trend. Proper Schedule H1 surveillance can help to identify the providers prescribing anti-TB drugs [43]. Tracking of the patient pathways, community surveys, and contextual enquiries can help in identifying the providers who could be targeted to address the ‘delay in diagnosis’ of TB.

**Segregated approach:** Specific strategies need to be in place to engage with all health care sectors outside of government, such as **(1)** corporate/ big hospitals, **(2)** clinics/ nursing homes/ individual practitioners, **(3)** chemists, **(4)** AYUSH practitioners, **(5)** informal health care providers, and **(6)** laboratories.

(1) **Corporate/big hospitals**: STEPS, which is envisioned as an equal partnership between the public and private sector for the benefit of society, where private hospitals establish systems to improve the quality of TB care, could be a good model for private hospitals [27]. PPSAs can support the establishing of STEPS in cities where private hospital concentration is higher. The NTEP can also advocate with the Insurance Regulatory and Development Authority of India (IRDAI), other private insurance agencies, and accreditation bodies such as the National Accreditation Board for Hospitals & Healthcare providers (NABH) to ensure that the patients getting treatment in private hospitals are being managed as per the STCI.
(2) **Individual medical practitioners**: They require access to opportunities for skill up-gradation and support for referrals, provision for access to molecular tests, supply of restricted medicines, support for documentation, patient support, feedback, and social recognition of their work [40,44]. A participatory program where a PPSA offers free access to rapid diagnostics, drugs, support for the notification of patients, and patient support has found to be effective in engaging private practitioners in Chennai [44]. Professional medical associations could play a major role in the capacity building of general practitioners and improving the quality of care through normative forces and peer pressure. Electronic learning courses with flexible schedules and provisions for periodically updating their knowledge through an established communication channel could be attempted. Social marketing of NTEP drugs and diagnostics might be helpful in increasing the uptake of these.
(3) **Chemists**: India has over 750,000 chemists and pharmacists who could be actively engaged in symptom screening, referral, and surveillance. Experiences from pharmacy engagement across the globe have revealed that establishing a system for simple screening among high-risk clients and a flexible referral mechanism at pharmacies is possible and has the potential to reduce the delay in diagnosis [45]. Chemists ‘associations and the Pharmacy Council of India need to be made stakeholders, and a system to train and update the knowledge of pharmacists needs to be established. Chemists also can play an important role in strengthening Schedule H1, which can help in identifying the right providers for engagement and strengthening surveillance. Schedule H1 has immense potential for identifying the right providers for engagement, strengthening the TB surveillance system, and improving the quality of care in the private sector. The NTEP needs to devise a strategy to strengthen Schedule H1 implementation by advocating with the drug control department and supporting them, establishing systems and tools for strengthening surveillance and collaborating with chemists individually and through their associations. Use of an API (application programming interface), which integrates billing software with Nikshay to generate real-time information of anti-TB drug sales, would have an immense potential to strengthen surveillance [43,46].
(4) **AYUSH:** Systematic training and sensitization for referral, extending the specimen collection and transportation mechanisms, and treatment support need to be conducted for AYUSH practitioners. Integrated delivery models such as hub-and-spoke models, where an AYUSH practitioner refers a patient for treatment initiation to a modern medicine practitioner/private health facility/government health facility, could be attempted.
(5) **Informal health care providers**: There is a growing recognition that it is essential to engage informal providers to reach out to all TB patients because of their significant presence in rural areas and also because they care for people in the lower socioeconomic strata who are disproportionately affected by the TB disease. The program needs to engage with the informal health care providers by educating them for symptom screening and referral, extending the specimen collection and transportation mechanisms, and empowering them as treatment supporters. There is limited experience of engaging informal health care providers for TB care [47,48]. Agencies with a deeper understanding of the community and its behavior need to be engaged to map the informal providers and systematically engage them. Enforcing the Schedule H1 regulations might help in reducing the number of unqualified practitioners prescribing anti-TB drugs.
(6) **Laboratory**: The NTEP needs to proactively improve the access to diagnostics for patients reaching the private sector through certifying more private laboratories for TB tests such as the line probe assay and DST, scaling up various models such as cartridge/chip sharing and reagent rental, or subsidizing rates through the IPAQT mechanism. The strategic purchase of services from private laboratories could improve the utilization and acceptability of DST among the private sector doctors.

**Change management and capacity building for NTEP officials and staff:** Behavior change strategies need to be devised to have a uniform outlook for state, district, and sub-district health officials with regard to private sector engagement. They need to clearly understand the national policies and strategies. The capacities of peripheral staff such as Public–Private Mix coordinators, TB health visitors, and senior treatment supervisors need to be built to deal with the private sector in a more efficient way.

**Strengthening platforms for engagement** Strengthening platforms, or structures for dialogue and communication between sectors, is important in building trust and the co-development of policies. Fostering relations allows stakeholders to move beyond simply understanding one another, to being able to work together. The Medical College Task Force is an example of a structured mechanism, where the NTEP interacts with all medical colleges [49]. It has state-, zonal-, and national-level structures. Similarly, a ‘Private Health Sector Task Force’ may be created with district-, state-, zonal-, and national-level structures.

**Expanding the social insurance coverage**: PM-JAY and other government insurance schemes should provide coverage to patients with pulmonary TB and expand their coverage to cover diagnostic and therapeutic procedures for all forms of TB. This will help in reducing the out-of-pocket expenditure for people with TB.

**Implementation models and documentation:** All the new interventions suggested need to happen in an implementation research mode with an intention to learn and quickly scale up the learnings.

## 5. Conclusions

India has taken good initiatives for engaging with the private health service sector through policy, regulation, partnerships, and financing. However, the scale, sustainability, and affordability of such initiatives remain major concerns. The country needs to consider an integrated private sector engagement policy, beyond a strategic purchase option. The policy action should match the total amount of engagement required, while taking into account the needs and behavior of the private health sector providers and those who seek their care. The country needs to have specific strategies to engage with all health care service providers other than those in the public sector, such as (1) corporate/ big hospitals, (2) clinics/ nursing homes/ individual practitioners, (3) chemists, (4) informal health care providers, (5) AYUSH practitioners, and (6) laboratories. Schedule H1 surveillance has immense potential in identifying the providers for engagement, strengthening the surveillance, and improving the quality of care. Documentation is essential to provide the necessary evidence to inform decision making.

## Figures and Tables

**Table 1:** Major strategies for private sector engagement for TB care in India.

Domains of Engagement	Strategies (Year of Initiation)	Description
Policy and dialogue	National Health Policy (NHP) 2017	NHP mentions about enabling the private sector contribution to make the health care systems more effective, efficient, rational, safe, affordable, and ethical [12].
	National Strategic Plan (NSP) for TB elimination from 2017 to 2025	NSP highlights the need for private sector engagement as an important component to eliminate TB [2].
	Standards of TB Care in India (STCI)	STCI mentions 26 standards that every citizen should receive irrespective of the sector of treatment [13].
Information Exchange	Ni-kshay (2012)	Ni-kshay is the real-time case-based web-based management information system of NTEP [14]. Private providers can directly log in to the system using their user credentials and notify TB and report outcomes. In total, 172,068 private hospitals were registered in Ni-kshay, out of which 36,346 notified at least one patient in 2021 [15].
	Incentive for TB notification and outcome reporting (2019)	NTEP provides INR 500 as an incentive to the private provider to notify each TB patient and another INR 500 to report the treatment outcome [16].
	Ni-kshay Sampark (2018)	Ni-kshay sampark is the national call center, which supports the private sector with notification and treatment adherence support. The program currently runs a 100-seat call center and supports 14 languages [15].
Regulatory Measures	Mandatory TB notification (2012)	The Government of India has issued directives making TB notification mandatory and promulgated another directive penalizing failure to notify [17].
	Enforcement of Schedule H1 regulation (2014)	Anti-TB medicines are included in Schedule H1 and can only be sold on prescription from a registered medical practitioner, and details of the prescriber, the patient, and the drug sold need to be maintained by the chemists [18]. This is intended to prevent the indiscriminate use of the drug.
	Price ceiling of anti-TB drugs	The National Pharmaceutical Pricing Authority (NPPA) has imposed a ceiling price for anti-TB drugs to be sold in the market [19].
Public Provision of Services	Free drugs and diagnostics (2017)	The NTEP has provisions for supplying free, quality-assured anti-TB drugs and free tests such as molecular tests and drug susceptibility tests to the patients reaching the private sector [2].
	Nikshay Poshan Yojana (2018)	The NTEP provides INR 500 per month to all TB patients, irrespective of sector, during the treatment period as a direct benefit transfer [15].
	Support in contact investigations, treatment adherence, and preventive therapy (2017)	Directly and through various agencies, the NTEP provides support for contact investigations and treatment adherence to all patients reaching private sector [2].
	Training and capacity building	Directly and through various agencies, the NTEP provides training and capacity building for the health workforce in the private sector.
Financing	National Partnership Guidelines (2019)	In 2001, the first guidelines on partnerships on engagement of non-governmental organizations (NGO) and private providers (revisions in 2008, 2014, and 2019) [20]. To increase the capacity of states for strategic purchase of services, multi-disciplinary technical support units have been formed in nine high priority States.
	Pradhan Mantri Jan Arogya Yojana (PM-JAY)	PM-JAY is a health insurance scheme that provides cashless cover of INR 500,000 per annum (USD 6700) to the eligible disadvantaged households for inpatient treatment at empaneled private hospitals for listed conditions which include management of patients with pleural, pericardial, and neuro-tuberculosis [21].
	Subsidy	The Initiative for Promoting Affordable and Quality Tests (IPAQT) in India provides over 130 accredited laboratories with concessionary pricing in exchange for their case notification to the NTP and passing on the price reductions to patients for WHO-endorsed TB tests [22].

**Table 2:** Trends in TB notifications and related indicators from the private sector in India, 2017–2021 [29].

	2017	2018	2019	2020	2021
TB Notification from the private sector	383,784	542,390	678,895	556,582	689,129
Proportion of TB notification from private out of the total notification	21%	25%	28%	31%	32%
Proportion of TB patients notified from private who knew their HIV status	NA	NA	55%	85%	92%
Proportion of notified TB patients from private offered universal drug susceptibility testing	NA	6%	28%	48%	39.4%
Treatment success rate of TB patients in the private sector	38%	35%	71%	79%	82%

## Data Availability

No new data were created for this study. Data sharing is not applicable to this article.
